# Idiopathic Slipped Capital Femoral Epiphysis: Demographic Differences and Similarities between Stable, Unstable, and Valgus Types

**DOI:** 10.3390/children10091557

**Published:** 2023-09-15

**Authors:** Randall T. Loder, Zachary Gunderson, Seungyup Sun

**Affiliations:** Department of Orthopaedic Surgery, Indiana University School of Medicine, Indianapolis, IN 46202, USA

**Keywords:** slipped capital femoral epiphysis, demographics, stable, unstable, valgus

## Abstract

Idiopathic slipped capital femoral epiphysis (SCFE) is a known disorder in pre/adolescent children with vague hip/knee pain. We wished to study the demographic differences between stable varus, unstable varus, and valgus idiopathic SCFEs using a retrospective review over a 10-year period of SCFE children seen at a tertiary children’s hospital. Standard demographic data was collected, and radiographs were measured to determine the Southwick angle and status of the tri-radiate cartilage. There were 190 patients; 138 had stable varus SCFEs, 45 unstable varus SCFEs, and 7 valgus SCFEs. All unstable SCFEs were varus, and all valgus SCFEs were stable. There were significant differences between the three groups by age at diagnosis, sex, race, SCFE severity, weight percentile, and duration of symptoms. The average age at diagnosis was 11.0 ± 1.2, 11.8 ± 1.8, and 12.3 ± 1.7 years for the valgus, unstable varus, and stable varus groups (*p* = 0.019), and similarly, SCFE severity was 25° ± 15°, 48° ± 18°, and 35° ± 19° (*p* = 0.0002) for the three same groups. Patients with valgus SCFEs were mostly female (86%) compared to the stable varus (39.9%) and unstable (47%) groups (*p* = 0.05) and mostly non-White (86%) (0.011). The duration of symptoms was 4.1 ± 4.1, 2.3 ± 5.0, and 4.5 ± 5.0 months for the valgus, unstable varus, and stable varus groups (*p* = 0.00005). These three types of idiopathic SCFEs demonstrated differences by age at diagnosis, sex, race, weight percentile, and duration of symptoms.

## 1. Introduction

Slipped capital femoral epiphysis (SCFE) can be divided into idiopathic and atypical types [1,2], with idiopathic being more common [1]. Most SCFEs demonstrate a varus deformity, although valgus types do exist [3,4,5]. The typical SCFE demonstrates an inferior displacement of the epiphysis relative to the metaphysis on radiographs, thus a varus deformity; however, a few may demonstrate a superior displacement of the epiphysis relative to the metaphysis on radiographs, or a valgus deformity. Similarly, they are either stable or unstable [6]. A stable SCFE is where the child can ambulate with or without crutches; an unstable SCFE is when the child can not ambulate, with or without crutches. [6].

The general demographics of SCFE have been relatively well studied [7,8,9,10,11,12,13,14,15,16,17,18,19]. There is a slight male predominance, with the most recent series in the 60% range for boys. The average age of presentation in modern-day children is 12 years for boys and 11 years for girls, but in studies from the early 1900s was 15 years [14]; the average symptom duration is 4 to 5 months. SCFE is relatively more common in Polynesian, Black, and Hispanic children compared to White children, and quite rare in those of Indian subcontinent descent [17]. Most of the children are overweight and/or obese. The vast majority of the SCFEs are the stable type. However, there are no studies comparing the demographics between all three types of idiopathic SCFEs—stable varus, unstable varus, and valgus. It was the purpose of this study to explore this area and see if differences exist in demographic variables between these three types of idiopathic SCFEs.

## 2. Materials and Methods

This is an observational study of all patients with SCFE treated at a tertiary children’s hospital for the time period January 2010 through March 2021. The medical records and radiographs were reviewed to confirm the diagnosis. Inclusion criteria were any patient with an idiopathic SCFE, excluding all non-idiopathic types. For patients with bilateral SCFEs, the data for the first hip was used for those with sequential presentation, and for those with bilateral simultaneous presentation, the hip having the longest duration of symptoms was used. The demographic data that was collected for this patient cohort was the child’s chronologic age, duration of symptoms, the height and weight of the patient at the time of diagnosis, and the child’s sex and race. For each of the SCFEs, the laterality (unilateral right or left, unilateral, sequential bilateral, and simultaneous bilateral). The stable/unstable nature of the SCFE [6] was determined from the medical records. The patient’s race was categorized as White or non-White. The lateral epiphyseal-shaft angle (LESA), as described by Southwick [20], was used to measure the severity of the SCFE. The SCFEs were designated as mild (<30° LESA), moderate (30–50° LESA), or severe (>50° LESA) [21]. The tri-radiate cartilage status at diagnosis of the first SCFE was graded as open, closing, or closed, as described by Acheson [22]. The senior author, with a long-standing interest in SCFE, reviewed the radiographs, performed the angular measurements, and determined the valgus/varus nature of the SCFE. The child’s height and weight were converted to percentiles using online growth charts from the CDC. This was performed using the SimulConsult app (https://simulconsult.com/resources/measurement.html?type=weight, accessed on 6 June 2021). The body mass index (BMI) (kg/m^2^) and percentile of the patient were calculated using the Baylor College of Medicine app (https://www.bcm.edu/cnrc-apps/bodycomp/bmiz2.html, accessed on 6 June 2021). Our local Institutional Review Board had approved the study.

Systat 10™ software was used to perform statistical analyses. Continuous variables are expressed as the average ± 1 standard deviation. Categorical variables are expressed as percentages and frequencies. Due to non-normal distributions of the continuous variables, differences between them were assessed using non-parametric statistics (for 2—variables, the Mann–Whitney U test was used; for 3 variables or more, the Kruskal–Wallis test was used). Categorical variable differences were assessed using the Fisher exact test for 2 × 2 analyses; Pearson’s χ^2^ test was used for when the analysis was greater than). A *p* < 0.05 was the threshold for statistical significance.

## 3. Results

**Figure 1:**
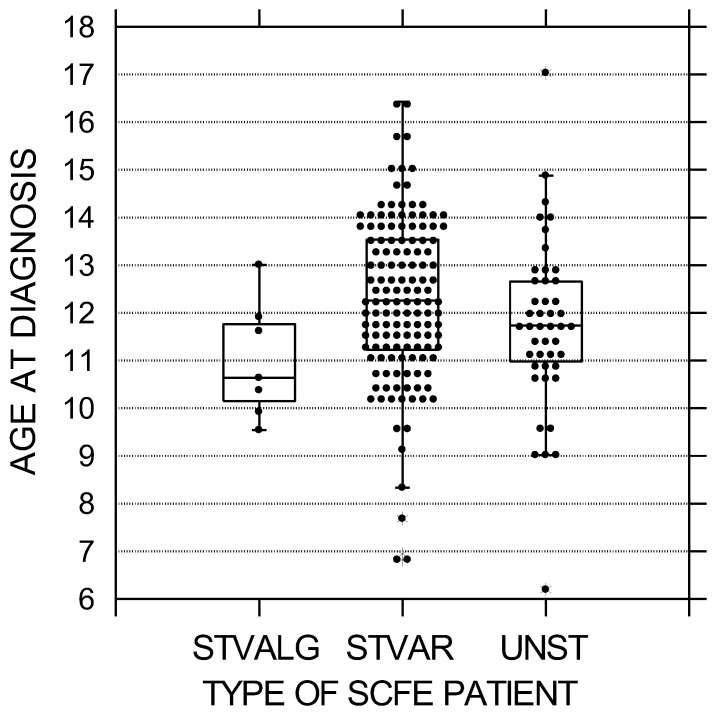
Age at diagnosis in years by SCFE type. The black circles represent each patient; the upper and lower boundaries of the boxes represent the upper and lower quartiles, while the bar inside the box is the median. These differences were statistically significant (*p* = 0.019). STVALG = stable valgus SCFE, STVAR = stable varus SCFE, UNST = unstable varus SCFE.

**Figure 2:**
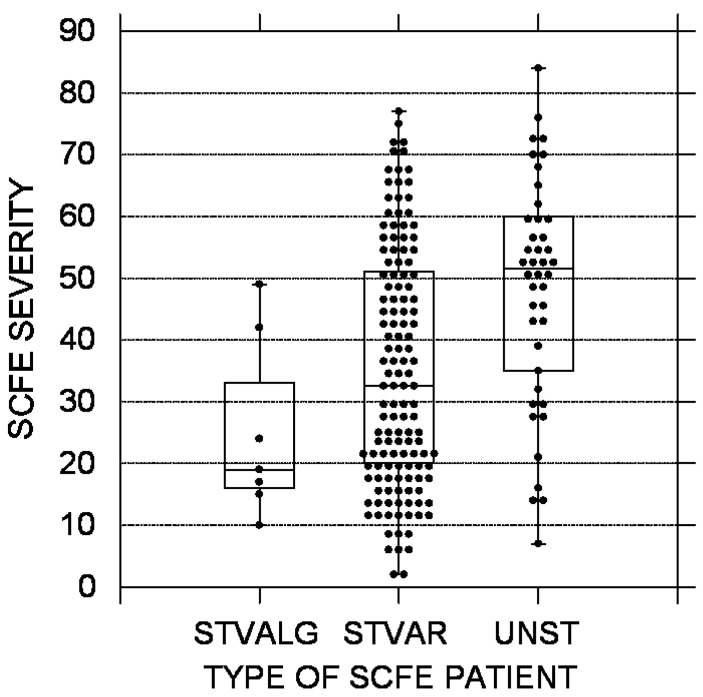
SCFE severity using the lateral epiphyseal shaft angle [20]. The black circles represent each patient; the upper and lower boundaries of the boxes represent the upper and lower quartiles, while the bar inside the box is the median. STVALG = stable valgus SCFE, STVAR = stable varus SCFE, UNST = unstable varus SCFE. These differences were statistically significant (*p* = 0.0002).

**Figure 3:**
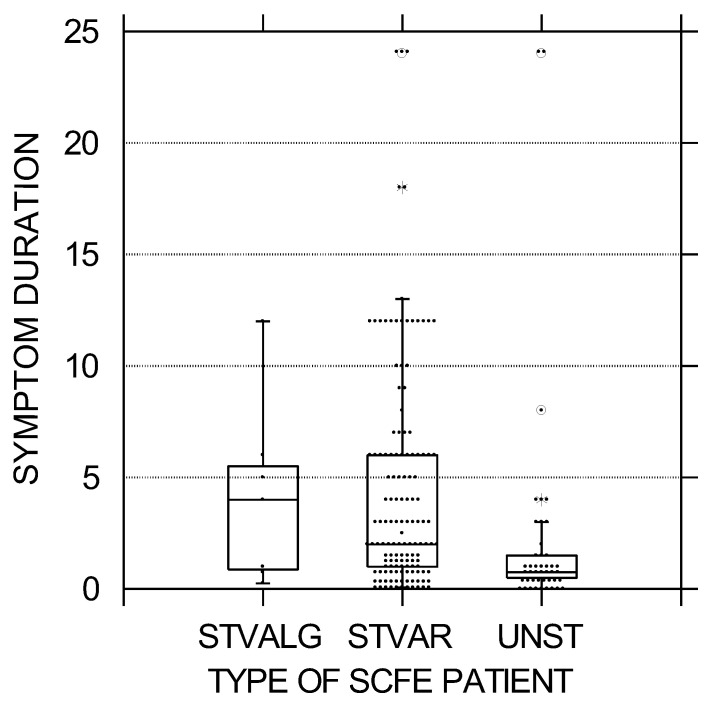
Symptom duration in months was less in the unstable SCFE group, and relatively equal between the stable varus and valgus groups. The black circles represent each patient; the upper and lower boundaries of the boxes represent the upper and lower quartiles, while the bar inside the box is the median. STVALG = stable valgus SCFE, STVAR = stable varus SCFE, UNST = unstable varus SCFE. These differences were statistically significant (*p* = 0.002).

In this cohort of 190 patients with 223 idiopathic SCFEs, 138 (72.6%) had stable varus SCFEs, 45 (23.6%) unstable varus SCFEs, and 7 (3.7%) valgus SCFEs. All unstable SCFEs were varus, and all valgus SCFEs were stable. Analyses between the three SCFE types (Table 1) demonstrated significant differences between all three groups by age at diagnosis, sex, race, SCFE severity, weight percentile, and duration of symptoms. Children with valgus SCFEs were the youngest and stable varus SCFEs the oldest (Figure 1). The average age for the valgus, unstable, and stable varus groups was 11.0 ± 1.2, 11.8 ± 1.8, and 12.3 ± 1.7 years, respectively (*p* = 0.019). Patients with valgus SCFEs were mostly female (86%—1 of 7) compared to the stable varus (39.9% 55 of 138) and unstable (47% 21 of 45) groups (*p* = 0.42). Children with valgus SCFEs were mostly non-White (86%—6 of 7) compared to the stable varus (39.4%—83 of 137) and the unstable (27%—12 of 44) (*p* = 0.011). SCFE severity was the lowest in the valgus group and highest in the unstable group (Figure 2). The average LESA for the valgus, unstable, and stable varus groups was 25 ± 15, 48 ± 18, and 35 ± 19, respectively (*p* = 0.0002) Weight percentile was lowest in the valgus group (82nd percentile) with the stable and unstable groups equal at 94th and 95th percentiles, respectively (*p* = 0.018). The duration of symptoms was less in the unstable group, and relatively equal between the stable varus and valgus groups, and was 2.3 ± 5.0 months in the unstable, 4.1 ± 4.1 months in the valgus, and 4.5 ± 5.0 months in the stable varus groups (*p* = 0.00005) (Figure 3). There were no differences in the ADI national percentiles between the three groups: 72 ± 20 in the stable varus, 71 ± 22 in the unstable varus, and 63 ± 31 in the stable valgus groups (*p* = 0.74).

Between the stable and unstable groups, the significant differences in age at diagnosis, SCFE severity, and symptom duration persisted. There were no differences by sex, race, or weight percentile. However, there was a difference in the status of the tri-radiate physis, being closed in 37% (51 of 138 patients) of the stable and 18% (8 of 45 patients) of the unstable SCFE patients (*p* = 0.018). Between the stable varus and stable valgus groups, the significant differences in age at diagnosis, race, and weight percentile persisted. There were no differences by SCFE severity or duration of symptoms.

**Figure 4:**
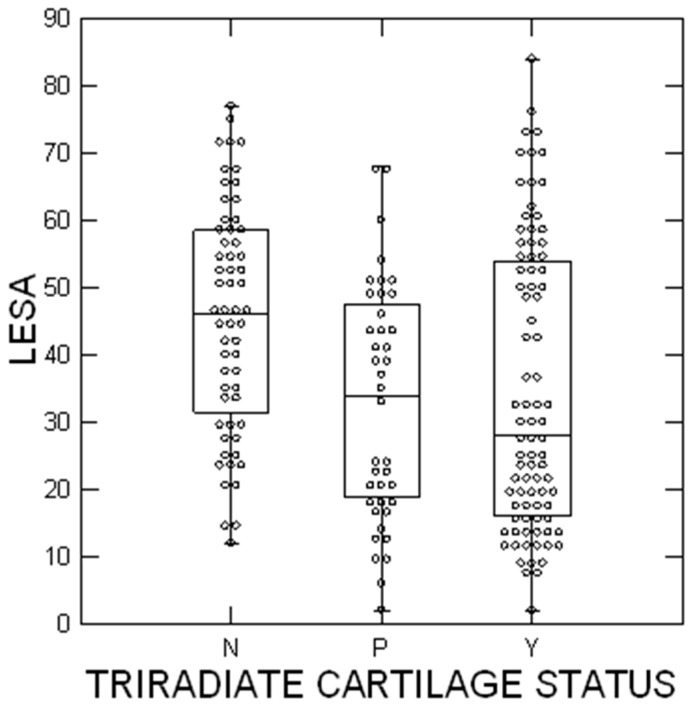
SCFE severity using the lateral epiphyseal shaft angle [20]. The circles represent each patient; the upper and lower boundaries of the boxes represent the upper and lower quartiles, while the bar inside the box is the median. N = tri-radiate cartilage not open, P = closing, and Y = open. These differences were statistically significant (*p* = 0.0004).

Further analyses were performed looking at the status of the tri-radiate physis (open, closing, and closed). This was performed for all 190 patients (Table 2) as well as for the stable SCFE group (Table 3). (Due to the smaller numbers of patients in the unstable and valgus groups, detailed analyses by tri-radiate physis status were not performed.) For the entire cohort, there was a significant difference by age at diagnosis, LESA, symptom duration, and laterality between the three groups. The average age was 11.4 ± 1.6, 12.0 ± 1.5, and 13.3 ± 1.3 years for the open, closing, and closed groups, respectively (*p* < 10^−6^). The average LESA was 35 ± 21, 33 ± 18, and 46 ± 17 for the open, closing, and closed groups, respectively (*p* = 0.0004) (Figure 4). The average duration of symptoms was 1.8 ± 2.4, 2.6 ± 2.4, and 7.7 ± 6.7 for the open, closing, and closed groups, respectively (*p* < 10^−6^). The SCFE was more commonly on the right for the closed group (57%) compared to the open (37%) and closing (47%) groups (*p* = 0.048). When analyzing only the stable SCFE group, the differences remained the same.

## 4. Discussion

This appears to be the first study comparing the demographics of idiopathic SCFE between all three types—stable varus, unstable, and valgus SCFEs. We noticed several interesting differences involving age at diagnosis, sex, race, SCFE severity, duration of symptoms, and body weight percentiles.

The age at diagnosis was significantly different between all three groups. The median age for the stable group was 12.3 years, 11.7 for the unstable group, and 10.6 for the valgus group. When comparing the literature studies (Table 2), a similar trend was noted for the unstable (12.3 years) and the stable group the oldest (12.8 years). In a meta-analysis of valgus SCFEs [3], the average age was 13.0 years; however, it must be noted that many of those studies were old, and it is known that there has been a gradual decrease in the age of all patients with SCFE over time. When using only studies from 2000 onward, the average age of the valgus groups was 12.2 years (Table 4). Thus, the differences in age at diagnosis between the three groups in our study (valgus youngest, stable oldest) are similar to those in the literature. These differences in age are also reflected in the status of the tri-radiate cartilage, with the unstable group having a higher percentage of patients with open tri-radiate cartilage (64%) compared to the stable varus (41.3%) and valgus (42%) groups.

There was a greater proportion of girls in the valgus group (86%) compared to 40% in the stable varus and 47% in the unstable varus groups. In a recent meta-analysis of 74 patients with valgus SCFEs, 62% were girls. This is much higher than the 35.7% female percentage in SCFE overall [17]. Thus, there clearly is a predilection for valgus SCFEs to be more common in girls. The exact reason for this is unknown, as there is minimal difference between sexes in the neck-shaft angle. Novais et al. [35] studied epiphyseal tilt with computed tomography of adolescents without hip pathology and found that females demonstrated slightly greater anterosuperior epiphyseal tilt (12.9° vs. 10.3°) and trended toward more superior tilt (*p* = 0.06) when compared to boys. Perhaps this puts the epiphysis in a slightly more valgus position, and when the appropriate stresses are placed on the proximal femur, a valgus SCFE occurs. Regarding the stable varus and unstable groups, there was a slightly higher percentage of girls in the unstable group (47% vs. 40%—*p* = 0.042). This is in agreement with a literature review where 46% of the unstable and 40% of the stable SCFEs were in girls [13].

Stable valgus SCFEs were much more common in non-White children than stable varus SCFEs (86% vs. 40%—*p* = 0.021). In an earlier study, spanning 1998 through 2003 from our institution [29], four patients with seven valgus SCFEs were described, and all were Black. In a recent study for Uruguay of eight children with valgus SCFEs [5], seven of the eight were White, and one was Black (Gelink A—personal communication). Segal et al. [4] described patients with valgus SCFE; one was Hispanic, and one was Black. Shank et al. [30] described 12 patients with valgus SCFEs; six were White, and five were Black. Yngve [28] described seven patients with valgus SCFEs; six were Black, and one was White. Koczewski [36] and Kalhor et al. [32] did not mention race in their studies. The racial makeup for these different studies of valgus SCFEs is quite mixed and may or may not represent the racial prevalence from that center or country in which the study originated. These are interesting findings for which we have no explanation, and which will require further study.

Unstable SCFEs had a larger LESA on average, compared to stable varus or valgus SCFEs. The average LESAs for these three groups in our study were 48°, 35°, and 25°, respectively. In a systematic review of 74 patients with valgus SCFEs [3], the average LESA was 23°. Interestingly, the valgus group had a longer duration of symptoms, but a lower LESA. Generally, a longer duration of symptoms is associated with greater LESA [34,37,38]. The average LESA in the literature for stable and unstable SCFE is 29° and 48°, respectively (Table 2), again very similar to our results. Perhaps valgus SCFEs are more stable biomechanically and do not progress as rapidly over time. This might be akin to the concept behind a valgus intertrochanteric osteotomy for the treatment of a femoral neck nonunion or congenital coxa vara.

The average duration of symptoms in patients with SCFE is often several months [13,14,33,39,40,41,42,43,44]. Most studies of SCFE do not differentiate the average duration of symptoms between stable and unstable types. Thus, it is difficult for us to find comparison studies in the literature. We found this surprising, as there are literally hundreds of SCFE papers since the 1993 stable/unstable classification was published, yet there are very few studies differentiating the demographics between the two groups. In this study, the average symptom duration for stable SCFEs was 4.5 months. In an older international study [13], the average duration of symptoms was 4.8 months for chronic SCFEs (which are typically all stable). In a multicenter study of only stable SCFEs [34], the average symptom duration was 5.2 months. In two recent studies, the average symptom duration was 5.2 months [33] and 4.1 months [37]. Both our results and those from the other studies are very similar. Regarding unstable SCFEs, the average symptom duration was less (2.3 months), indicating that there were precedent symptoms before the event leading to the unstable SCFE. This has been previously noted by McPartland et al. [27] in 82 patients with unstable SCFEs; 88% had a history of prior symptoms with an average of 1.4 months. Our 2.3 months is longer than the 1.4 months in the study of McPartland et al. [27], but still with the same conclusion that unstable SCFEs usually have precedent symptoms.

There was an overall difference between the three groups by weight percentiles; however, it was only true between the stable varus and valgus groups (Table 1). The stable varus weight percentile was 95th, the unstable varus 94th, and the valgus group 82nd. A previous study noted that non-obese patients were more likely to present with an unstable SCFE [45] using BMI as the obesity measurement. In our study, we found no difference in the weight percentile between the stable and unstable groups. The Obana study [45] excluded patients if they had no recorded height and weight; the number excluded was not given. This makes comparisons difficult. When reviewing the data from our study where there was a height and thus a BMI, we noted no differences in BMI percentile between the three groups. The patient’s height was not known in all our cases, reflecting our center’s philosophy of intake with any new SCFE patient. When the diagnosis is known ahead of time before an outpatient visit, a height is not obtained as that requires the patient to stand, which is not desired. Thus, our nurses have been instructed to forgo obtaining a height. The emergency department is also a common entry point for SCFE patients, and frequently, the intake personnel do not obtain or record the patient’s height.

It is well known that an open tri-radiate physis indicates a younger patient, and that was confirmed in this study. Information regarding the status of the tri-radiate physis in SCFE patients has been primarily used when considering prophylactic fixation of the opposite hip when a patient with a unilateral SCFE presents. Popejoy et al. [36] were instrumental in pointing this out, and the literature regarding the status of the tri-radiate physis in SCFE patients seems to concentrate solely on the consideration for prophylactic fixation of the opposite hip [46,47,48,49,50,51,52,53,54,55]. Our findings that the status of the tri-radiate physis in SCFE patients is also correlated with SCFE severity (LESA) is likely new and needs further corroboration from other centers.

The strength of this study is that it represents the demographics of SCFE from one tertiary children’s hospital, comparing all three types of idiopathic SCFE, which has not been previously conducted. The discussion above compares either one type from different centers or two types from one center; this study compares all three types from a single center, eliminating the potential bias when comparing different studies. There are certain limitations to this study. The height was only available in 90 of the 138 patients, not allowing us to calculate a BMI in every patient. With retrospective studies, symptom duration is dependent upon patient recall and may not be completely accurate. Racial identification was patient-determined. There were many patients having Hispanic surnames, but they self-registered as White. Thus, some of those in the White group may actually be Hispanic/persons of color and thus not White. Finally, the number of valgus SCFE cases was low, similar to all valgus SCFE studies. However, the non-parametric statistics did demonstrate differences in certain areas (e.g., weight percentile and age), indicating that these differences were real, in spite of the small number of valgus cases.

## 5. Conclusions

This study has demonstrated that in the three types of idiopathic SCFE, there are differences by age at diagnosis, sex, race, weight percentile, and duration of symptoms. This is baseline data for further studies of children with SCFE, and we encourage researchers in the future to state what type(s) of SCFE is being studied, as there are many differences between these three different types.

## Figures and Tables

**Table 1:** Demographic data by type of SCFE.

	All	Stable Varus	Unstable Varus	Stable Valgus	*p*-Value ^$^	*p*-Value *	*p*-Value ^^^
**Continuous variables**							
**All**	190	138	45	7	-	-	-
**LESA (mean ± 1 sd)**	38 ± 20	35 ± 19	48 ± 18	25 ± 15	0.0002	0.0002	0.17
**Age**							
**Mean ± 1 sd**	12.1 ± 1.7	12.3 ± 1.7	11.8 ± 1.8	11.0 ± 1.2	0.019	0.048	0.027
**Median (range)**	12.0 (6.2–17.0)	12.3 (6.8–16.4)	11.7 (6.2–17.0)	10.6 (9.5–13.0)			
**Weight percentile**	94 ± 10	95 ± 9	94 ± 9	82 ± 18	0.018	0.081	0.014
**Height percentile**	75 ± 26	76 ± 25	76 ± 24	64 ± 41	0.84	0.86	0.56
**BMI percentile**	93 ± 15	93 ± 16	90 ± 11	94 ± 9	0.30	0.12	0.97
**Symptom duration (mos)**	3.9 ± 5.0	4.5 ± 5.0	2.3 ± 5.0	4.1 ± 4.1	0.00005	0.000009	0.92
**ADI national percentile**	72 ± 21	72 ± 20	71 ± 22	63 ± 31	0.74	0.67	0.47
**Categorical variables**							
**Sex**							
**Female**	82 (43.2)	55 (39.9)	21 (47)	6 (86)	0.05	0.49	0.042
**Male**	108 (56.8)	83 (60.1)	24 (53)	1 (14)			
**Race**							
**Non-white**	72 (38.3)	54 (39.4)	12 (27)	6 (86)	0.011	0.16	0.021
**White**	116 (61.7)	83 (60.6)	32 (73)	1 (14)			
**Laterality**							
**Left**	103 (54.2)	74 (53.6)	25 (56)	4 (57)	0.96	0.86	1.00
**Right**	87 (45.8)	64 (46.4)	20 (44)	3 (43)			
**Tri-radiate cartilage**							
**Closed**	61 (32.1)	51 (37.0)	8 (18)	2 (29)	0.082	0.018	0.87
**Closing**	40 (21.1)	30 (21.7)	8 (18)	2 (29)			
**Open**	89 (46.8)	57 (41.3)	29 (64)	3 (42)			

The numbers in parentheses for the categorical variables are column percentages. ^$^ *p* value between all three groups, * *p* value between the stable and unstable groups, ^^^ *p* value between the stable varus and stable valgus groups.

**Table 2:** Status of triradiate cartilage by demographic variables.

	Open	Closing	Closed	*p*-Value ^$^	*p*-Value *	*p*-Value ^^^
	89	40	61	-	-	-
**LESA (mean ± 1 sd)**	35 ± 21	33 ± 18	46 ± 17	0.0004	0.74	0.0007
**Age**						
**Mean ± 1 sd**	11.4 ± 1.6	12.0 ± 1.5	13.3 ± 1.3	<10^−6^	0.069	<10^−6^
**Median (range)**	11.5 (6.2–14.4)	11.8 (8.3–15.0)	13.2 (11.2–17.0)			
**Weight percentile**	94 ± 9	94 ± 11	95 ± 11	0.89	-	-
**Height percentile**	77 ± 26	75 ± 29	73 ± 25	0.42	-	-
**BMI percentile**	93 ± 16	93 ± 14	92 ± 16	0.73	-	-
**Symptom duration (mos)**	1.8 ± 2.4	2.6 ± 2.4	7.7 ± 6.7	<10^−6^	0.006	<10^−6^
**ADI national percentile**	73 ± 20	66 ± 26	73 ± 17	0.47	-	-
**Sex**						
**Female**	34 (38)	22 (55)	26 (43)	0.20	-	-
**Male**	55 (62)	18 (45)	35 (57)			
**Race**						
**Non-white**	29 (33)	14 (35)	29	0.19	-	-
**White**	58 (67)	26 (65)	32			
**Laterality**						
**Left**	56 (63)	21 (53)	26 (43)	0.048	0.33	0.019
**Right**	33 (37)	19 (47)	35 (57)			

The numbers in parentheses for the categorical variables are column percentages. ^$^ *p* value between all three groups, * *p* value between the open and closing groups, ^^^ *p* value between the open and closed groups.

**Table 3:** Status of triradiate cartilage by demographic variables for the stable SCFE group.

	Open	Closing	Closed	*p*-Value ^$^	*p*-Value *	*p*-Value ^^^
	57	30	51	-	-	-
**LESA (mean ± 1 sd)**	28 ± 19	28 ± 16	46 ± 17	<10^−6^	0.94	<10^−6^
**Age**						
**Mean ± 1 sd**	11.5 ± 1.6	12.0 ± 1.6	13.3 ± 1.3	<10^−6^	0.62	0.01
**Median (range)**	11.5 (6.8–14.4)	11.8 (8.3–15.0)	13.3 (11.2–16.4)			
**Weight percentile**	96 ± 7	95 ± 11	95 ± 10	0.91	-	-
**Height percentile**	78 ± 26	78 ± 28	72 ± 22	0.15	-	-
**BMI percentile**	93 ± 17	94 ± 15	93 ± 16	0.57	-	-
**Symptom duration (mos)**	2.3 ± 2.8	2.8 ± 2.6	7.8 ± 6.1	<10^−6^	0.76	<10^−6^
**ADI national percentile**	74 ± 20	69 ± 24	73 ± 16	0.83	-	-
**Sex**						
**Female**	18 (32)	17 (57)	29 (48)	0.075	-	-
**Male**	39 (68)	13 (43)	31 (52)			
**Race**						
**Non-white**	19 (37)	10 (33)	25 (49)	0.21	-	-
**White**	37 (63)	20 (67)	26 (51)			
**Laterality**						
**Left**	38 (67)	14 (47)	22 (43)	0.034	0.33	0.019
**Right**	19 (33)	16 (53)	29 (57)			

The numbers in parentheses for the categorical variables are column percentages. ^$^ *p* value between all three groups, * *p* value between the open and closing groups, ^^^ *p* value between the open and closed groups.

**Table 4:** Synopsis of the literature.

Author	Year	Overall Number in the Study	Number of Appropriate Patients within the Subgroup ^&^	Average Age (Years)	Average LESA *	Symptom Duration (Months)	Boys	Girls	%Girls
Unstable SCFEs									
Loder [6]	1993	NA	30	12	51	NA	14	16	53.3
Kalogrianitis [23]	2007	82	16	12.3	NA	NA	9	7	43.8
Chen [24]	2009	NA	23	11.9	NA	1.0	16	7	30.4
Palocaren [25]	2010	280	27	12.2	51	NA	19	8	29.6
Alves [26]	2012	189	12	12.2	33	NA	6	6	50.0
McPartland [27]	2013	582	82	12.5	NA	1.4	41	41	50.0
Weighted average		1133	190	12.3	48		105	85	44.7
Valgus SCFEs									
Yngve [28]	2005	NA	7	14.1	34	NA	3	4	57.1
Loder [29]	2006	105	4	11.7	14	13.0	2	2	50.0
Shank [30]	2010	258	12	11.6	30	1.8	5	7	58.3
Koczewski [31]	2013	115	11	11.1	23	2.7	5	6	54.5
Kalhor [32]	2018	NA	6	13.8	21	NA	3	3	50.0
Gelink [5]	2020	NA	8	11.9	28		2	6	75.0
Weighted average			48	12.2	26		20	28	58.3
Loder [13]	1996	1630	1363	12.9	NA	4.8	812	551	40.4
Stable SCFEs									
Hosseinzadeh [33]	2017	NA	149	11.8	NA	5.2	89	60	40.3
Loder ^^^ [34]	2006	NA	243	12.6	29	5.2	159	84	34.6
Weighted average				12.8	29		1060	695	39.6

* LESA = the lateral epiphyseal shaft angle of Southwick [20]; ^^^ using the older terminology of chronic, which is in all likelihood stable; ^&^ denotes the number of cases in the study that fit the SCFE type; NA means the data was not available.

## Data Availability

The data presented in this study are available on request from the corresponding author. The data are not publicly available due to the need to access HIPAA-protected medical records.
